# Injuries from falls by older adults in long-term care captured on video: Prevalence of impacts and injuries to body parts

**DOI:** 10.1186/s12877-022-03041-3

**Published:** 2022-04-19

**Authors:** Vicki Komisar, Aleksandra Dojnov, Yijian Yang, Nataliya Shishov, Helen Chong, Ying Yu, Ian Bercovitz, Michael D. Cusimano, Clemens Becker, Dawn C. Mackey, Stephen N. Robinovitch

**Affiliations:** 1 Department of Biomedical Physiology and Kinesiology, Simon Fraser University, 8888 University Drive, Burnaby, BC V5A 1S6 Canada; 2 School of Engineering, The University of British Columbia, 1137 Alumni Ave, Kelowna, BC V1V 1V7 Canada; 3 Institute of Biomedical Engineering, University of Toronto, 164 College Street, Toronto, ON M5S 3G9 Canada; 4 Department of Sports Science and Physical Education, The Chinese University of Hong Kong, SAR, Shenzhen, China; 5 Department of Statistics and Actuarial Science, Simon Fraser University, 8888 University Drive, Burnaby, BC V5A 1S6 Canada; 6 Division of Neurosurgery, St. Michael’s Hospital, Unity Health, LiKaShing Knowledge Institute, 209 Victoria St, Toronto, ON M5B 1T8 Canada; 7 Robert Bosch Hospital, Auerbachstraße 110, 70376 Stuttgart, Germany; 8 Unit of Digital Geriatric Medicine, University Hospital, Im Neuenheimer Feld 672, 69120 Heidelberg, Germany

**Keywords:** Falls, Older Adults, Injury, Bone fracture, Head injury, Residential care, Nursing homes, Video capture and analysis

## Abstract

**Background:**

Falls are the leading cause of injuries in older adults. However, most falls in older adults do not cause serious injury, suggesting that older adults may fall in a manner that reduces the likelihood of impact to body sites that are most vulnerable to injury. In this observational study of falls in long-term care (LTC), we tested whether body parts differed in their probability of impact and injury.

**Methods:**

We recorded and analyzed videos of 2388 falls by 658 LTC residents (mean age 84.0 (SD = 8.1); 56.4% female). We used Linear Mixed Models to test for differences between body parts in the probability of impact and injury, and injury when impacts occurred.

**Results:**

Injuries were reported in 38.2% of falls, and 85.9% of injuries involved direct impact to the injured body part. Impact occurred most often to the hip/pelvis (probability (standard error) = 0.95 (0.01); *p* < .001 relative to other body parts), and least often to the head (0.35 (0.01)). Conversely, injury occurred most often to the head (*p* < .001 relative to other body parts). The probability of injury when impacts occurred was 0.40 (0.01) for the head, and 0.11 or less for all other body parts.

**Conclusion:**

Our results help to explain why most falls by older adults in LTC do not cause serious injury: residents land on body parts that are the most resilient to injury. The high susceptibility of the head to injury reinforces the need to enhance upper limb protective responses for fall arrest. The dominant role of direct impact as the mechanism of injury supports approaches to attenuate impact forces through strategies like protective clothing and compliant flooring.

## Background

Falls exert a tremendous health toll on older adults, defined as those 65 years and older. Falls cause over 90% of hip fractures [1] and up to 80% of TBIs in older adults [2, 3], and are a leading cause of trauma-related hospitalizations and a top ten cause of death [4, 5]. Older adults often limit their mobility and physical activity due to fear of falling, which can also compromise their health and wellbeing [6, 7].

At the same time, it is important to consider that, from a physical trauma perspective, most falls in older adults do not result in serious injury. Between 20–30% of falls cause some type of injury [8, 9], and 2–5% lead to hospital visits [10, 11]. Injuries from falls in older adults are a serious health problem not because every fall is catastrophic, but rather because falls are so frequent. Approximately 30% of older adults who are living independently and up to 60% living in long-term care (LTC) will fall at least once per year, and many will fall repeatedly [12, 13].

An important question for injury prevention is understanding how the risk for injury during a fall depends on characteristics of the fall, and on tissue strength in resisting trauma [14–16]. For example, previous studies have found that the risk for hip fracture depends as much on the mechanics of the fall (falling sideways, and landing on the hip) as it does on bone density [17, 18]. Subsequently, balance assessment techniques have targeted lateral stability [19], and wearable hip protectors have emerged as a valuable tool for reducing the risk for hip fracture among older adults who are willing to wear them [20]. Further improvements in injury prevention may be informed by a more comprehensive understanding of the how the mechanics of falls influence the spectrum of injuries caused by falls. However, we lack objective evidence on the circumstances of falls, to compare with injury patterns. Our understanding is based largely on the self (or witness) reports on fall circumstances, which are prone to bias and inaccuracy [21]. Two notable exceptions are Parkkari et al.’s study of hematoma patterns in falls causing hip fracture [22], and our recent study of video-captured falls causing hip fracture [23].

The current study expands the evidence base by linking injuries to the landing patterns of falls captured on video in two partnering long-term care homes in the Vancouver area [23, 24]. We focused specifically on determining how injury risk depended on direct impact to the injured body part (via contact with the ground or an object in the environment), versus propagation of forces from the impact site to the site of injury (e.g., shoulder injury from bracing of the fall with the outstretched hand). We examine whether our results support the notion that older adults tend to avoid impact to body parts that are most vulnerable to injury, as a possible explanation for why most falls in older adults do not cause substantial physical trauma. This notion is well-supported in young adults, who coordinate their protective responses (e.g., upper limb fall arrest) to avoid or reduce impacts to the head and pelvis during falls [25, 26]. However, there is no evidence on whether older adults in LTC, many of whom have dementia and physical frailty, coordinate their body movements during falls to avoid impacts to vulnerable body parts [27]. We hypothesized that: (1) body parts differ in their probability of experiencing direct impact with the ground or an object in the environment during falls; (2) body parts differ in their frequency of experiencing injury in falls; and (3) the risk for injury to body parts during falls is increased by direct impact of that body part with the ground or an object in the environment.

## Methods

### Participants and care setting

From January 2010 to September 2019, we collected and analyzed video footage and corresponding fall incident reports of 2388 real-life falls by 658 residents who were 65 years or older, residing in one of two LTC homes in the Greater Vancouver Area (New Vista, a 236-bed site in Burnaby, BC, and Delta View, a 312-bed site in Delta, BC) [28]. This study was conducted in accordance with the Declaration of Helsinki. This study was approved by the Office of Research Ethics at Simon Fraser University (approval number H21-00,741), and also reviewed and approved by the Fraser Health Authority, and the Behavioural Research Ethics Board of the University of British Columbia – Okanagan Campus. Each resident or proxy decision maker provided written informed consent for the recording of video in common areas (e.g., dining rooms). Video footage and fall incident reports were shared as secondary data with our research team. We also analyzed a subset of falls where we obtained separate written informed consent from participants, or their proxy decision makers, to access their medical records. No cameras were in bedrooms or bathrooms. The videos had a resolution of at least 640 × 480 pixels, and a frame rate of 15–30 Hz.

### Video coding

Videos of falls were analyzed by teams of three trained raters, who reviewed video footage of each fall, and completed a structured, validated questionnaire to classify features of the initiation, descent, and impact stages of the fall [29]. In this study, we considered the presence of impact with the ground or an object in the environment (e.g., tables or chairs) to the head, torso/shoulder, hip/pelvis, knee/shin, elbow/forearm, and hand/wrist. The reliability of these measures is previously documented [29].

### Injury data

Injury outcomes were based on fall incident reports (completed by LTC staff) and review of medical records by Simon Fraser University researchers. At the commencement of the study, we worked with both LTC homes to integrate information on injuries into fall incident reports, including the location of injury (e.g., head, torso, pelvis, lower extremities, upper extremities) and the type of injury, which was classified as: (1) fracture, (2) sprain, strain or dislocation, (3) cut, scrape or abrasion, (4) bruise, bump, redness or swelling, and (5) pain, with or without palpation. The accuracy of injury data provided on the incident report was confirmed, and adjusted as necessary, based on review of medical records for the 7-day period after the fall. We defined “serious” injuries as those that either prompted a visit to the hospital or medical clinic, or suturing [30]. All other injuries were defined as “minor” [30].

### Statistics

We used binary logistic regression linear mixed models (MIXED Procedure, SAS Version 9.4, Cary, NC) to test for differences between body parts in their probability of experiencing impact (hypothesis 1; model 1), and in their probability of experiencing injury (hypothesis 2; model 2). In these models, impact was coded as yes versus no, and injury was coded as yes versus no. In model 2, we included body part impact as an explanatory variable, to test whether risk for injury to body parts was increased by impact to that body part (hypothesis 3). We also ran separate models to test for differences between body parts in the risk for serious injury (versus minor or no injury) in falls (model 3). To account for lack of independence between repeated falls in a given resident, and between impacts to multiple body parts in a given fall, we included resident identification codes and fall identification codes as random effects. We included sex (male versus female) and age (younger than the median value of 85 years, versus 85 years or older) in our models, given their documented association with fall-related injuries [23, 24, 31]. Where significant main effects and interactions were identified, we performed post hoc pairwise comparisons. We also performed secondary analyses to examine how the odds for hip/pelvis injury associated with use of wearable hip protectors (as noted in fall incident reports). A significance level of α = 0.05 was used for all analyses.

## Results

### Resident characteristics

2388 falls by 658 residents (ages 65 years and older) were captured on video. Participants had a mean age of 84.0 years (SD = 8.1 years), a median age of 85 years, and included 372 women (56.4%) and 286 men (43.6%). Of the residents who consented to accessing medical records via the Minimum Data Set (*n* = 260; 39%), 57.3% were dependent in ADL performance, and 67.7% had moderate to severe cognitive impairment (Table 1). Relative to residents who avoided injury in some or all falls on video, residents who were injured in all falls had higher BMI and body mass (*p* ≤ 0.003). They were also 2.2-fold less likely to have more advanced cognitive impairment (odds ratio = 2.20; 95% CI = 1.57–3.08), and 2.2-fold less likely to have an Alzheimer’s disease diagnosis (OR = 2.22; 95% CI = 1.60–3.08). We found no further differences in health status and medication use, and no differences in age and sex, between residents who were and were not injured in falls.

**Table 1:** Characteristics of the 260 participants who provided consent to access medical records

	Baseline value of residents with falls on video (*n* = 260)	Residents without injuries in any fall on video (*n* = 86)	Residents with injuries in all falls on video (*n* = 62)	Residents with and without injuries from falls on video (*n* = 112)	P*
**Demographics and health status**					
Age, mean (SD)^a^	84.1 (7.8)	84.1 (7.8)	85.1 (8.1)	83.7 (7.4)	.544
Female, n (%)^b^	149 (57.3)	46 (53.5)	34 (54.8)	69 (61.6)	.469
Height (cm), mean (SD)	163.2 (10.8)	163.4 (9.4)	164.9 (11.6)	162.0 (11.2)	.246
Body mass (kg), mean (SD)	62.5 (16.0)	63.7 (15.8)^B^	67.9 (17.8)^B^	58.4 (13.9)^A^	** < .001**
BMI (kg/m^2^), mean (SD)	23.4 (5.2)	23.7 (5.3)^AB^	25.0 (5.9)^B^	22.2 (4.5)^A^	**.003**
Dependent ADL^c^ performance, n (%)	149 (57.3)	52 (60.5)	31 (50.0)	66 (58.9)	.402
Moderate to severe cognitive impairment ^d^, n (%)	176 (67.7)	61 (71.8)	33 (53.2)*	81 (72.3)	**.022**
**Disease diagnoses, n (%)**					
Diabetes	60 (23.1)	17 (19.8)	17 (27.4)	26 (23.2)	.551
Cardiac dysrhythmia	15 (5.8)	4 (4.7)	6 (9.7)	5 (4.5)	.318
Congestive heart failure	20 (7.7)	8 (9.3)	6 (9.7)	6 (5.4)	.468
Hypertension	129 (49.6)	38 (44.2)	39 (62.9)	52 (46.4)	.054
Hypotension	12 (4.6)	2 (2.3)	5 (8.1)	5 (4.5)	.259
Alzheimer’s disease	66 (25.3)	24 (27.9)	8 (12.9)*	34 (30.4)	**.033**
Stroke	39 (15.0)	18 (20.9)	7 (11.3)	14 (12.5)	.166
Parkinson’s disease	10 (3.8)	4 (4.7)	2 (3.2)	4 (3.6)	.888
Emphysema / COPD^e^	30 (11.5)	11 (12.8)	6 (9.7)	12 (11.6)	.842
**Use of medications, n (%)**					
Antipsychotics	106 (40.8)	33 (38.4)	22 (35.5)	51 (45.5)	.372
Antianxiety agents	48 (18.5)	17 (19.8)	8 (12.9)	23 (20.5)	.430
Antidepressants	125 (48.1)	40 (46.5)	32 (51.6)	53 (47.3)	.810
Hypnotics	54 (20.8)	21 (24.4)	13 (21.0)	20 (17.8)	.529
Diuretics	51 (19.6)	18 (20.9)	17 (27.4)	16 (14.3)	.105
Analgesics	129 (49.6)	38 (44.2)	37 (59.7)	54 (48.2)	.164

^*^statistical comparisons between the resident injury groups (without injuries, with injuries, mix of injury and no injuries) were performed with Chi-square tests for categorical variables, and a 1-way ANOVA for age (continuous variable). Where significant main effects were identified, pairwise comparisons between residents without injuries, residents with injuries, and residents with and without injuries caught on video, were performed

^a^Age data were available for the entire sample of 658 residents; the mean (SD) resident age was 84.0 (8.1) years

^b^Sex data were available for the entire sample of 658 residents; fall data from 372 women (56.4%) were analysed

^c^ADL – “Activities of Daily Living”; scores of 0–2 were classified as “independent”; scores of 3–6 were classified as “dependent” on care staff

^d^CPS – “Cognitive Performance Scale”; scores of 0–2 were classified as “intact to mild cognitive impairment”; scores of 3–6 were classified as “moderate to severe cognitive impairment”

^e^COPD – “Chronic Obstructive Pulmonary Disease”

### Injury characteristics

**Fig. 1:**
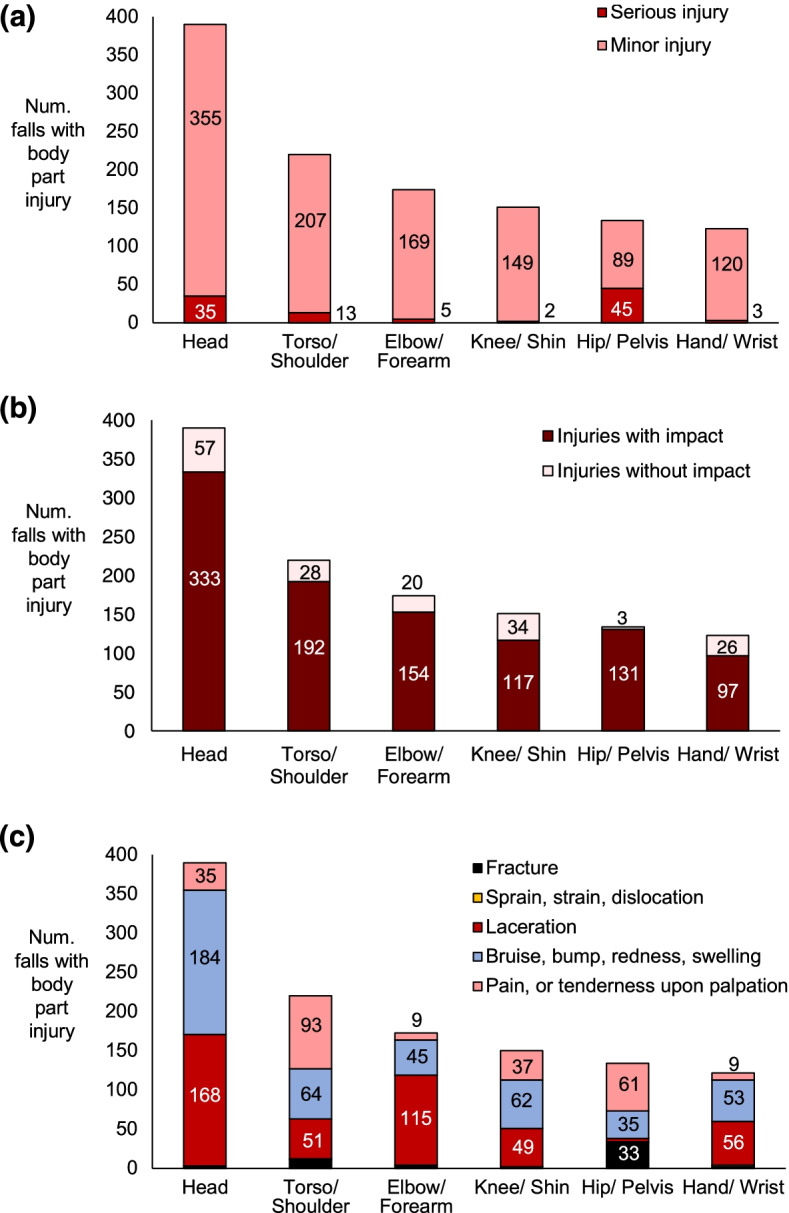
Injury distribution by body part and **a** injury severity, **b** occurrence of impact to the injured body part, and **c** injury type. In **a**, numbers to the right of the bars near the horizontal axis indicate the number of severe injuries to the body part. In **c**, there were also 3 fractures to the head, 1 torso/shoulder sprain, strain or dislocation (SSD), 11 torso/shoulder fractures, 3 hip/pelvis lacerations, 1 hip/pelvis SSD, 2 knee/shin SSDs, 1 unspecified knee/shin injury, 4 elbow/forearm fractures, 1 unspecified elbow/forearm injury, 2 hand/wrist SSDs, 2 hand/wrist fractures, and 1 unspecified hand/wrist injury

Of the 2388 analyzed falls, no injury was reported for 1476 falls (61.8%), 912 falls (38.2%) caused at least one documented injury, and 99 falls (4.2%) caused serious injuries. Injury was documented to only one body part in 678 falls (28.4% of falls), to two body parts in 167 falls (7.0% of falls), and to three or more body parts in 67 falls (2.8% of falls). Consequently, the total number of injuries to different body parts (*n* = 1216 for any injury; *n* = 103 for serious injuries) exceeded the total number of falls. We excluded 24 injuries from statistical analysis (14 to the ankle, and 10 to unspecified locations) because data were not available on the occurrence of impact to the injured body part. Accordingly, our statistical analysis included 1192 injuries to different body parts (Fig. 1).

### Probability of impacts to body parts

**Fig. 2:**
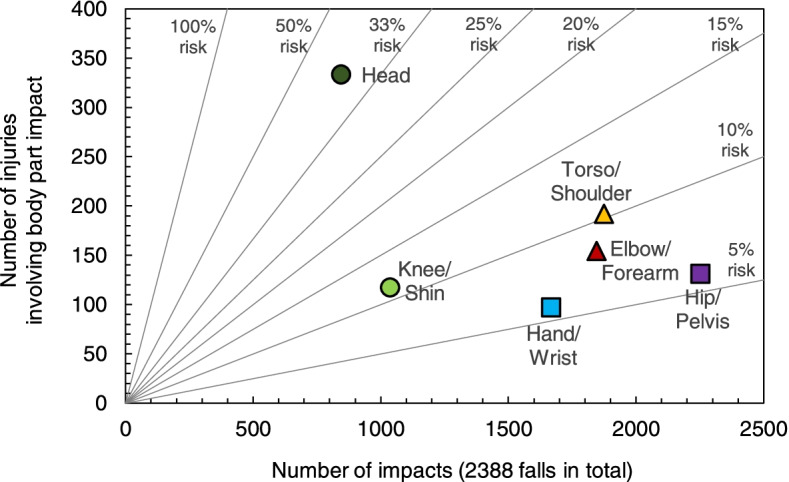
Distribution by body part in the prevalence of impact to the ground or an environmental object, and the prevalence of injury in the event of impact. Diagonal lines show the risk for injury in the event of impact

Body parts differed in their probability of impact (*p* < 0.001; Table 2 and Fig. 2). The highest probability for impact was to the hip/pelvis (0.95 (0.01)), followed by the torso/shoulder (0.79 (0.01)), elbow/forearm (0.78 (0.01)), hand/wrist (0.71 (0.01)), knee (0.44 (0.01)), and head (0.35 (0.01)). Pairwise comparisons revealed differences between all body parts in the probability for impact (*p* ≤ 0.001), except for the torso/shoulder and the elbow/forearm (*p* = 0.345).

**Table 2:** Estimated probability of body part impacts: least square mean estimates, standard errors (SE), and pairwise comparisons

Body part	All falls, impact probability (SE) *n* = 2388	Resident age			Resident sex		
		Youngest half, impact probability (SE) *n* = 1197	Oldest half, impact probability (SE) *n* = 1191	*P*_Age_	Men, impact probability (SE) *n* = 1029	Women, impact probability (SE) *n* = 1359	*p*_*sex*_
Head	0.35 (0.01)^E^ *n* = 846	0.35 (0.01)^E^ *n* = 415	0.36 (0.01)^E^ *n* = 431	.787	0.30 (0.01)^E^ *n* = 301	0.40 (0.01)^D^ *n* = 545	** < .001**
Torso/ shoulder	0.79 (0.01)^B^ *n* = 1875	0.78 (0.01)^B^ *n* = 922	0.80 (0.01)^B^ *n* = 953	.186	0.78 (0.01)^B^ *n* = 786	0.80 (0.01)^B^ *n* = 1089	.153
Hip/ pelvis	0.95 (0.01)^A^ *n* = 2253	0.95 (0.01)^A^ *n* = 1127	0.95 (0.01)^A^ *n* = 1126	.984	0.94 (0.01)^A^ *n* = 957	0.96 (0.01)^A^ *n* = 1296	.427
Knee/ shin	0.44 (0.01)^D^ *n* = 1038	0.46 (0.01)^D^ *n* = 545	0.42 (0.01)^D^ *n* = 493	**.032**	0.48 (0.01)^D^ *n* = 481	0.41 (0.01)^D^ *n* = 557	** < .001**
Elbow/ forearm	0.78 (0.01)^B^ *n* = 1846	0.78 (0.01)^B^ *n* = 924	0.78 (0.01)^B^ *n* = 922	.928	0.77 (0.01)^B^ *n* = 785	0.78 (0.01)^B^ *n* = 1061	.620
Hand/ wrist	0.71 (0.01)^C^ *n* = 1668	0.73 (0.01)^C^ *n* = 860	0.68 (0.01)^C^ *n* = 808	**.025**	0.71 (0.01)^C^ *n* = 726	0.70 (0.01)^C^ *n* = 942	.347

Superscripts by impact probability (SE) values indicate statistical comparisons between body parts for the column of interest. Body parts that differed significantly (*p* < .05) in impact probability are denoted by different letters; body parts where the probability of impact did not differ significantly (*p* > .05) are indicated by the same letter. The letter sequence is from highest to lowest least-square means

### Probability of injuries to body parts

Body parts differed in their probability of injury (*p* < 0.001). The most common site for injury was the head, which occurred in 390 falls (16.0% of all falls, and 42.8% of the 912 falls with injuries; Fig. 1a). The head was more likely to be injured than all other body parts (probability = 0.22 (0.01) versus 0.08 (0.01) for the torso/shoulder, 0.07 (0.01) for the knee and elbow/forearm, 0.05 (0.01) for the hand/wrist, and 0.04 (0.01) for the hip/pelvis). The most common type of injury was bruising for the head and knee, lacerations for the elbow/forearm and hand/wrist, and pain for the torso/shoulder and hip/pelvis (Fig. 1c).

### Influence of body part impact on probability of injury

Of the 1192 documented injuries, 1024 injuries (85.9%) involved impact to the injured body part to the ground on an object in the environment, and 168 injuries (14.1%) did not involve impact to the injured body part. Body parts differed in their probability of injury in the event of impact (*p* < 0.001; Table 3 and Fig. 2). The highest probability of injury in the event of impact was for the head (0.40 (0.01)), followed by the torso/shoulder and knee (0.11 (0.01)), elbow/forearm (0.09 (0.01)), and hand/wrist and hip/pelvis (0.06 (0.01)). The probability of injury increased with impact for all body parts except the hip/pelvis (*p* = 0.101) and the hand/wrist (*p* = 0.088). When impacts did not occur, there were no differences between body parts in the probability of injury (*p* ≥ 0.053).

**Table 3:** Estimated probability of body part injuries: least square mean estimates, standard errors (SE), and pairwise comparisons

Body part	All falls, injury probability (SE) *n* = 2388	Body part impact			Resident age			Resident sex		
		Impact to body part, injury probability (SE)	No impact to body part, injury probability (SE)	*p*_*mpact*_	Younger half, injury probability (SE) *n* = 1197	Older half, injury probability (SE) *n* = 1191	*p*_Age_	Men, injury probability (SE) *n* = 1029	Women, injury probability (SE) *n* = 1359	*p*_*sex*_
Head	0.22 (0.01)^A^ *n* = 390	0.40 (0.01)^A^ *n* = 333	0.05 (0.01)^A^ *n* = 57	** < .001**	0.21 (0.01)^A^ *n* = 179	0.23 (0.01)^A^ *n* = 211	**.032**	0.22 (0.01)^A^ *n* = 142	0.22 (0.01)^A^ *n* = 248	.959
Torso/ shoulder	0.08 (0.01)^B^ *n* = 220	0.11 (0.01)^B^ *n* = 192	0.06 (0.01)^A^ *n* = 28	** < .001**	0.08 (0.01)^B^ *n* = 103	0.09 (0.01)^B^ *n* = 117	.554	0.08 (0.01)^B^ *n* = 91	0.09 (0.01)^B^ *n* = 129	.732
Hip/ pelvis	0.04 (0.01)^C^ *n* = 134	0.06 (0.01)^D^ *n* = 131	0.02 (0.01)^A^ *n* = 3	.101	0.04 (0.01)^C^ *n* = 60	0.05 (0.01)^C^ *n* = 74	.707	0.03 (0.01)^D^ *n* = 45	0.05 (0.01)^CD^ *n* = 89	.087
Knee/ shin	0.07 (0.01)^B^ *n* = 151	0.11 (0.01)^B^ *n* = 117	0.04 (0.01)^A^ *n* = 34	** < .001**	0.07 (0.01)^B^ *n* = 75	0.08 (0.01)^B^ *n* = 76	.624	0.07 (0.01)^BC^ *n* = 62	0.08 (0.01)^BC^ *n* = 89	.629
Elbow/ forearm	0.07 (0.01)^BC^ *n* = 174	0.09 (0.01)^C^ *n* = 154	0.05 (0.01)^A^ *n* = 20	**.001**	0.06 (0.01)^B^ *n* = 81	0.07 (0.01)^BC^ *n* = 93	.471	0.08 (0.01)^B^ *n* = 90	0.06 (0.01)^CD^ *n* = 84	**.037**
Hand/ wrist	0.05 (0.01)^C^ *n* = 123	0.06 (0.01)^D^ *n* = 97	0.04 (0.01)^A^ *n* = 26	.088	0.04 (0.01)^C^ *n* = 44	0.07 (0.01)^BC^ *n* = 79	**.016**	0.05 (0.01)^CD^ *n* = 53	0.05 (0.01)^D^ *n* = 70	.832

Superscripts by injury probability (SE) values indicate statistical comparisons between body parts for the column of interest. Body parts that differed significantly (*p* < .05) in injury probability are denoted by different letters; body parts where the probability of injury did not differ significantly (*p* > .05) are indicated by the same letter. The letter sequence is from highest to lowest least-square means

### Influence of age

There was a significant interaction between age and body part on probability for impact (*p* = 0.021). Older residents were less likely to experience knee and hand/wrist impact than younger residents (*p* ≤ 0.032; Table 2). There was also a significant main effect of age on probability for injury (*p* = 0.048). Older residents were more likely than younger to experience injuries during falls (0.10 (0.01) versus 0.08 (0.01)), particularly to the head and hand/wrist (*p* ≤ 0.032; Table 3).

### Influence of sex

There was a significant interaction between sex and body part on probability for impact (*p* < 0.001; Table 2). Women were more likely than men to experience head impact (0.40 (0.01) versus 0.30 (0.01); *p* < 0.001), while men were more likely to experience knee impact (0.48 (0.01) versus 0.41 (0.01); *p* < 0.001). The interaction between sex and body part on probability for injury approached significance (*p* = 0.069; Table 3). Pairwise comparisons revealed that men were more likely to experience elbow/forearm injury (0.08 (0.01) versus 0.06 (0.01); *p* = 0.037).

### Serious injuries

Body parts differed in their probability of serious injury (*p* < 0.001). The hip/pelvis and the head had the highest probability of serious injury (0.020 (0.002) and 0.016 (0.002) versus ≤ 0.007 for other body parts). 45 falls involved serious injuries to the hip/pelvis (1.9% of all falls), of which 33 were fractures (Fig. 1a). 35 falls involved serious injuries to the head (1.5% of all falls), of which 13 cases involved lacerations requiring sutures; 4 cases involved loss of consciousness; 3 involved a confirmed skull fracture; and 2 involved a confirmed intracranial hemorrhage. Impact to the head accompanied 94.3% of serious injuries to the head, and impact to the hip/pelvis occurred in 100% of falls causing serious hip/pelvis injuries. 3.9% of head impacts caused serious head injury, and 2.0% of hip/pelvis impacts caused serious hip/pelvis injury. For all other body parts, serious injuries occurred in < 0.7% of impacts to the body part. Older residents were more likely to sustain serious injuries to any body part (0.011 (0.001) versus 0.007 (0.001); *p* = 0.035). Older and younger residents experienced serious head injuries in 1.7% and 1.3% of all falls, and serious hip/pelvis injuries in 2.4% and 1.4% of all falls, respectively. Sex was not significantly associated with risk for serious injury in falls (*p* = 0.079).

### Influence of hip protector use during falls

Residents wore hip protectors in 72% of falls in this study, which likely contributed to the overall resilience of the hip/pelvis in resisting impact-related injuries. We found no effect of hip protector use on the likelihood of hip/pelvis impact in this study (*p* = 0.245 by Chi-square analysis). However, residents were twofold less likely to experience a hip/pelvis injury when impacts occurred when wearing hip protectors (odds ratio = 0.47; 95% confidence interval = 0.32–0.69). When hip/pelvis impact occurred, residents experienced hip/pelvis injuries in 9.4% of cases without hip protector use, and in 4.7% of cases when hip protectors were worn.

## Discussion

We provide novel video-based evidence of the landing patterns of falls in older adults, and the relationship between impact and injury to different body parts. We found that 86% of injuries were associated with impact of the injured body part with the ground or an object in the environment, as opposed to being caused by the propagation of forces from the impact site to a different site of injury (e.g., shoulder or elbow injuries from bracing of the fall with the hands [32–34]). These results agree with previous studies showing that traumatic brain injuries were associated with a history and signs of head impact, and that fractures of the hip, wrist and skull associated with impact to those body parts [17, 22, 23, 35, 36]. The dominant role of direct impact as the cause of injuries supports strategies to reduce or prevent injuries in LTC through protective clothing [20, 23, 37], compliant (safety) flooring [30, 38], mobility aids [39], and exercises to enhance protective responses for arresting falls [40].

We observed a statistically significant effect of impact on risk for injury for all body parts, with the notable exception of the hip/pelvis and hand/wrist. The lack of association between impact and injury for the hip/pelvis and hand/wrist probably relates to the high probability for impact, and low probability for injury in the event of impact to these body parts. Indeed, the large majority of injuries to these body parts were associated with impact, including 100% of serious hip/pelvis injuries. However, the probability for minor injury to the hip/pelvis and hand/wrist was just as high in the small number of falls that did not involve impact to these body parts. For the hand/wrist, non-impact injuries may have occurred from grasping or contact of the hand on nearby objects (furniture, walkers, wheelchairs, or walls). For the hip/pelvis, non-contact injuries may have resulted from force transmission during knee impact.

Our results show that, regardless of sex or age, the body parts with the highest susceptibility to injury (in the event of impact) were the least likely to experience impact, while the body parts that were most resilient to injury were the most common sites of impact. While the hip/pelvis was the most likely of all body parts to experience direct impact to the ground or an object in the environment during a fall (at 95% probability), it was the least likely of all body parts to be injured in the event of impact (at 6%). The head was the least likely of all body parts to experience impact during a fall (at 35% probability), and the most likely to be injured in the event of impact (at 40%). The trends were similar for serious injuries, which occurred for 4% of impacts to the head, 2% of impacts to the hip/pelvis, and < 0.7% of impacts to other body parts. Our findings suggest that older adults fell in a manner that protected against impact to body parts that were the most vulnerable to injury (i.e., the head), and increased their likelihood for impact to body parts that were more resistant to injury (e.g., the hip/pelvis).

The locations and types of injuries we observed were aligned to previous reports on injuries from falls by older adults who reside in LTC [41–44] and in the community [45, 46]. A Finnish LTC study [41] reported injuries in 38.1% of falls, including head injuries in 19.3% of falls, and fractures in 3.1% of falls, most often to the hip (1.5% of falls). In a Canadian geriatric rehabilitation unit, injuries occurred in 39.3% of falls [47]. In studies of falls in Swedish [43] and Bavarian [42] nursing homes, injuries occurred in approximately 25% of falls, with the latter reporting hospitalizations in 7.6% of falls in common spaces. Bruising/hematoma, followed by abrasions/cuts, were the most commonly reported injuries from falls in community-dwelling older adults in the Netherlands and United States [45, 46], and in LTC facilities in Sweden and China [43, 44].

Among the 39% of participants who provided us with access to medical records, we found few differences in clinical status between those who did and did not experience injury during falls. The notable exception was for cognitive status, where we found that participants who were more cognitively impaired were less likely to experience injury in a fall. Similar results have been observed for falls in the hospital setting, where patients with dementia were 1.3-fold less likely to be injured during a fall [48]. The difference may relate to the tendency for falls among cognitively impaired residents to occur more often during sitting or transferring as opposed to walking [24], or to under-detection of injuries in cognitively impaired individuals secondary to challenges in communicating pain [49].

The only difference we observed between men and women in risk for injury to specific body parts during falls was a slightly higher risk among men for injury to elbow/forearm. Surprisingly, there were no differences in risk for head injury, despite women having a significantly higher prevalence of head impact in falls. The trends suggest that, when head impact did occur, it was more likely to cause injury in men than women. Previous studies have reported conflicting results on sex-based differences in injury risk during falls. Gryfe et al. found no sex-based differences in injuries from falls in LTC [12]. Teo et al. found no sex-based differences in the frequency of fall-related TBI among older adults in the community [50], while other studies are divided between those that reported a greater incidence of fall-related TBI [51, 52] or head injuries in general [41, 53] in men, and those that reported a higher incidence of fall-related TBI [3, 54] or head injury hospitalizations [31, 55] in women. Buchele et al. [42] found that serious injuries were 1.3-fold more likely in women than men in LTC, driven largely by hip fractures. Our study may have lacked the statistical power to detect the size of the effect observed by Buchele et al. [42].

When compared to younger residents (< 85 years old – the median resident age), older residents were less likely to impact their knee and hand/wrist during falls, and more likely to experience injury to the head and hand/wrist. The higher prevalence of injuries in older residents is consistent with previous evidence of increases with age in the risk for injury from a fall [12, 42, 54, 55]. The absence of age-related differences in the probability of head impact suggests that strategies for avoiding head impacts did not diminish in effectiveness with age. The higher incidence of injuries to the head and hand/wrist suggests that, when impact to these body parts did occur, it was either more severe, or more likely to exceed diminished injury thresholds.

Residents wore hip protectors in 72% of falls in our sample, and the odds for hip/pelvis injury were reduced by two-fold during protected falls. Hip/pelvis injuries occurred in 9.4% of falls where hip protectors were not used, and where impact occurred to the hip/pelvis. This is still well below the 40% probability of head injuries when head impacts occurred. While the hip/pelvis sustained the highest number of fractures and serious injuries, these occurred in 2.0% of impacts, compared to serious head injuries reported in 3.9% of impacts. Together, these findings indicate that, even without the use of hip protectors, the hips/pelvis was more resilient than the head to experiencing impact-related injuries.

This study has important strengths. By linking injury patterns to video evidence of the landing patterns of real-life falls in older adults, we overcome the limitations of previous studies that relied on the questionable accuracy of self-described reports on the circumstances of falls [56]. In particular, our approach allowed us to compare body parts in terms of the probability for impact, and the probability for injury in the event of impact.

Our study also has several limitations. First, we examined whether there were differences between body parts in the frequency of impact and injury during falls. Several additional variables that were beyond our scope to explore may affect risk for injury during falls, including the severity of impacts, the direction of impact forces, and tissue tolerances for injury. Second, we determined injury outcomes from review of fall incident reports and medical records for 7 days after the fall. We restricted our review period to 7 days to reduce the likelihood of erroneously assuming that signs and symptoms (such as pain or bruising) were caused by the fall in question. However, this may have caused us to miss some injuries that resulted from the falls we examined. For example, patients can develop subdural hematoma weeks or months after a fall event [57]. Third, pre-existing cognitive impairment was common in our study cohort (cognitive impairment was rated as moderate to high in 67.7% of residents who provided us with access to medical records; Table 1), and may have created challenges in identifying the neurologic consequences of falls, including traumatic brain injury. Fourth, our video analysis focused on falls by LTC residents in common spaces (dining halls, lounges, and hallways). Falls in the common spaces we examined may differ from falls in bathrooms or bedrooms, or from falls on stairs, curbs or irregular terrain [48, 58–60]. Injury patterns are also likely to differ in LTC sites that do not have similar rates of use of mobility aids (37%), which provide a twofold reduction in the risk for head impact in falls [39]. Finally, we examined how injury risk associated with the occurrence of impact to body parts, but we did not measure the severity of impacts, based for example on impact velocity. Further work is needed to understand how injury risk depends on detailed kinematic characteristics of falls [61, 62].

## Conclusion

Our study shows that injuries from falls in LTC are usually associated with direct impact of the injured body part with the ground or an object in the environment. Furthermore, older adults in LTC fell in a manner that reduced the likelihood of head impact, which was the most vulnerable site for injury in the event of impact irrespective of age or sex. The hip/pelvis was 2.7 times more likely than the head to experience impact, and 6.7 times more resistant to injury than the head in the event of impact. Women were more likely to experience head impact during falls, but were no more likely than men to experience head injury. When compared to younger residents, older residents were no more likely to experience head impacts, but were more likely to experience head injuries. Our results help to explain why most falls by older adults in LTC do not result in serious injury: residents land on body parts that are the most resilient to injury. The high susceptibility of the head to injury reinforces the need for preventative strategies such as to enhance upper limb protective responses for safely arresting falls [40]. The dominant role of direct impact as the mechanisms of injury supports approaches to attenuate impact forces through protective clothing, compliant flooring, mobility aids, and exercise [30, 37–39].

## Data Availability

The datasets analysed during the current study are available from the corresponding author on reasonable request.
